# Hypoxia pathway and hypoxia-mediated extensive extramedullary hematopoiesis are involved in ursolic acid's anti-metastatic effect in 4T1 tumor bearing mice

**DOI:** 10.18632/oncotarget.12375

**Published:** 2016-09-30

**Authors:** Jian-Li Gao, Yan-Mei Shui, Wei Jiang, En-Yi Huang, Qi-Yang Shou, Xin Ji, Bai-Cheng He, Gui-Yuan Lv, Tong-Chuan He

**Affiliations:** ^1^ Zhejiang Chinese Medical University, Hangzhou, 310053, China; ^2^ Molecular Oncology Laboratory, The University of Chicago Medical Center, Chicago, IL 60637, USA; ^3^ Chongqing Medical University, Chongqing 400016, China; ^4^ School of Medicine, Zhejiang University, Hangzhou, 310058, China

**Keywords:** breast cancer, metastasis, hypoxic pathway, extensive extramedullary hematopoiesis, ursolic acid

## Abstract

Hypoxic in the tumor mass is leading to the myeloproliferative-like disease (leukemoid reaction) and anemia of body, which characterized by strong extensive extramedullary hematopoiesis (EMH) in spleen. As the key transcription factor of hypoxia, hypoxia-inducible factor-1 (HIF-1) activates the expression of genes essential for EMH processes including enhanced blood cell production and angiogenesis. We found ursolic acid (UA), a natural pentacyclic triterpenoid carboxylic acid, inhibited growth of breast cancer both *in vivo* and *in vitro*. The suppression was mediated through the inhibition of multiple cell pathways linked to inflammation, proliferation, angiogenesis, and metastasis. UA also suppressed the leukemoid reaction and the EMH phenomenon of the tumor bearing mice without any significant suppression on body weight (*i*.*p*. by 20 mg/kg for 28 days). This is associated with the significant decrease in white blood cells (WBC), platelets (PLT) and spleen weight. During this process, we also detected the down-regulation of cell proliferative genes (PCNA, and β-catenin), and metastatic genes (VEGF, and HIF-1α), as well as the depression of nuclear protein intensity of HIF-1α. Furthermore, the expression of E2F1, p53 and MDM2 genes were increased in UA group when the VEGF and HIF-1α was over-expressed. Cancer cells were sensitive to UA treating after the silencing of HIF-1α and the response of Hypoxic pathway reporter to UA was suppressed when HIF-1α was over expressed. Overall, our results from experimental and predictive studies suggest that the anticancer activity of UA may be at least in part caused by suppressing the cancer hypoxia and hypoxia-mediated EMH.

## INTRODUCTION

Breast cancer is the most common solid malignancy among women (almost 26.8%–29% in all cancer cases), both in the United States [1] and in China [2]. In 2013, 85% breast cancer cases were invasion cases in younger women under age 40 years [3]. Thus, in order to increase survival, methods of prevention and treatment of cancer metastases are necessary.

The close link between hypoxia and metastasis has been well recognized. Hypoxia affects both metastatic spread and selection of aggressive cells both in experimental and clinical studies [4]. Actually, tumor hypoxia is associated with many steps in cancer, such as angiogenesis, metastasis, and dysregulated hematopoiesis [5–7]. In mouse breast tumor, hypoxia-responsive pathways modulate extensive extramedullary hematopoiesis (EMH) and angiogenesis [8, 9].

Hypoxia-inducible factor (HIF-1) is one of the principal regulators in hypoxia-responsive pathways. It plays a pivotal role in hypoxia-mediated physiological responses and should be a remarkable target for developing novel cancer therapeutics. Indeed HIF-1α inhibitor has been confirmed a positive effect for depressing tumor growth and metastasis [10]. Another hypoxia factor HIF-2α was also associated with tumor induced EMH, it could induce erythropoietin (EPO) production and mediate tumor-related splenic erythropoiesis [11].

Ursolic acid (UA), a pentacyclic triterpene acid, is a versatile compound derived from many fruits like apples, pears, prunes and loquat, as well as other plants such as: bearberries (*Arctostaphylos alpina*), rosemary (*Rosemarinus officinalis*), cranberries (*Vaccinium macrocarpon*), makino (*Perilla frutescens*), scotch heather (*Calluna vulgaris*), basil (*Ocimum sanctum*), *Satureja khuzistanica* and jamun (*Eugenia jumbolana*). UA has a potential in prevention of cancer [12, 13], has been shown to suppress the growth of various tumors, including breast carcinoma, lung cancer, prostate cancer and colorectal carcinoma [14–18].

The objective of our study was to investigate the *in vitro* anticancer effects of UA on different breast cancer cell lines (67NR, MCF7, UACC3199, HCC38, HCC1937, MDA-MB-361, MDA-MB-435, SK-BR-3 and HS578T). The anti-tumor growth and metastasis efficacy of UA and its mechanism in tumor bearing mice were also detected using bioluminescence imaging (BLI) at different time points following 4T1-Luc tumor cell introduction. We have recently shown that UA is a potent inhibitor of hypoxia pathway, which suppressed hypoxia and Hypoxia-mediated EMH by inhibiting HIF-1α and VEGF (vascular endothelial growth factor) expression, but up-regulated the VHL (von Hippel-Lindau), E2F1, p53 and MDM2 gene expression.

## RESULTS

### UA reveals cytotoxicity towards several human cancer cells

**Figure 1:**
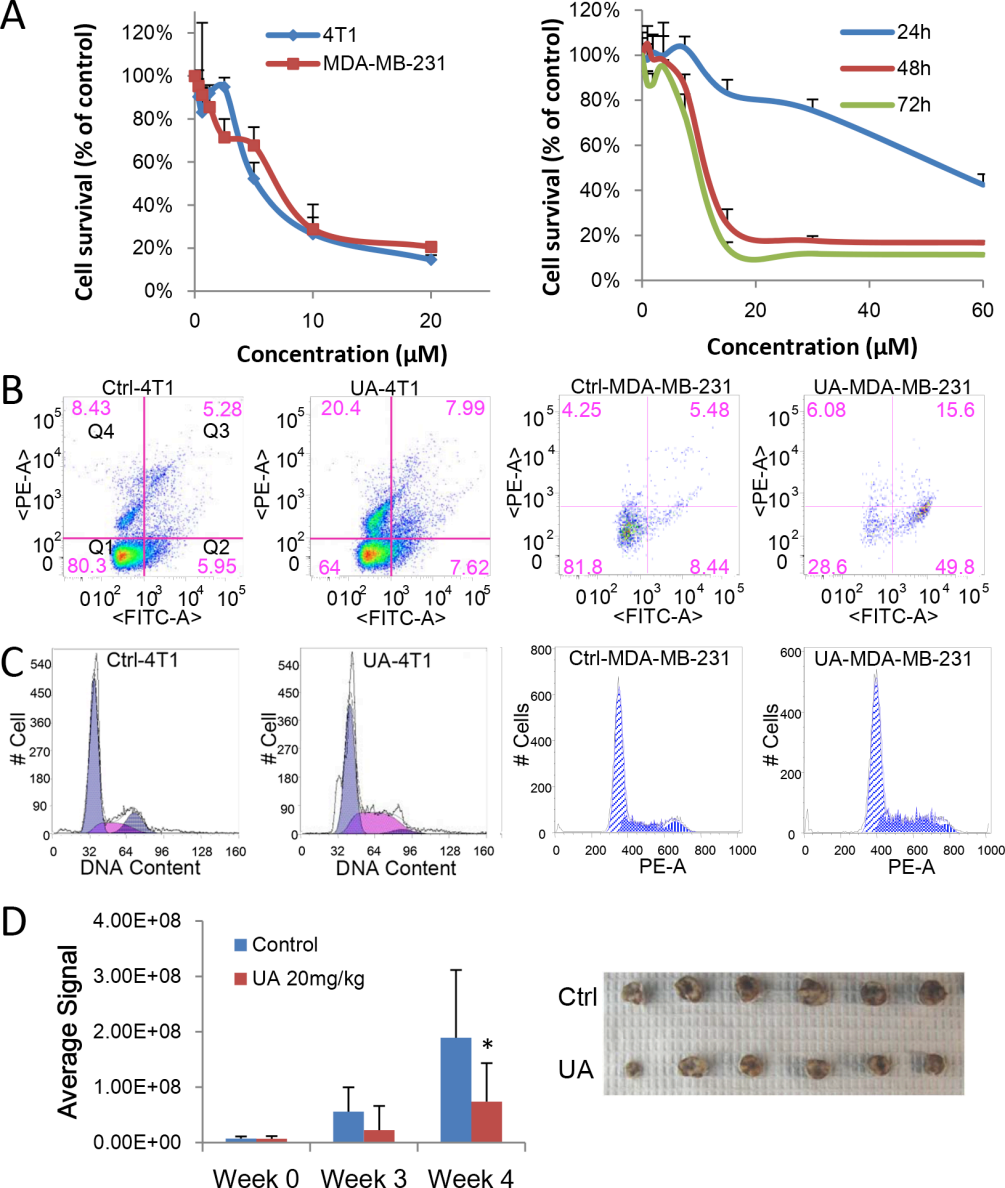
UA inhibits proliferation of breast cancer *in vitro* and *in vivo* (**A**) MTT assay. Left panel: 4T1 or MDA-MB-231 cells were treated with different concentration of UA for 48 hours; Right panel: MDA-MB-231 cells were treated with different concentration of UA for 24, 48 or 72 hours. (**B**) Cell apoptosis assay. 4T1 or MDA-MB-231 cells treated with 30 μM UA for 24 h. Cells were collect with trypsin without EDTA, labeled with Annexin V-EGFP and PI and then analyzed on flowcytometer. Dual parameter dot plot of FITC-fluorescence (x-axis) versus PI fluorescence (y-axis) has been shown in logarithmic fluorescence intensity. Quadrants: Q1 = live cells; Q2&Q3 = apoptotic cells; Q4 = necrotic cells. (**C**) Cell cycle analysis. 4T1 or MDA-MB-231 cells were treated with 10 μM UA for 48 h. Then cells were fixed and nuclear DNA was labeled with PI. Cell cycle distribution was analyzed by flowcytometry. Histogram display of DNA content (x-axis [PE-A]: PI-fluorescence) versus cell counts (y-axis) has been shown. (**D**) Left panel: Quantitative analysis of Xenogen imaging signal intensity (photons/sec/cm^2^/steradian) after 3 and 4 weeks treatment with UA; *P < 0.05. Right panel: UA reduced the primary tumor size (top panel, control; bottom panel, UA group). Tumor bearing mice was treated with 20 mg/kg UA and sizes of tumors derived from 4T1 cells were compared after 4 weeks.

Several researches have been reported the cytotoxicity of UA [19–21], but it is still unclear that how UA's anticancer activity is compared with chemotherapy drugs commonly used in clinical. We tested the antiproliferative activity of UA and six chemotherapy drugs in MDA-MB-468 and MDA-MB-231 (human breast cancer), HCT116 and SW480 (colon cancer), PC3 and DU145 (prostate cancer), MG63 and 143B (osteosarcoma). The calculated IC50 was ≤ 10 μM for UA in tested lines with the exception of MG63. It was lower than 5-FU and carboplatin, and similar with adriamycin (ADR), camptothecin, vincristine, and paclitaxel in most cell lines (Table 1). Moreover, UA was demonstrated to have a time-dependent and dose-dependent manner on 4T1 and MDA-MB-231 cells (Figure 1A). It is strongly suggested that UA exhibits strong cytotoxicities (IC50 from 2 to 20 μM) against a board range of human cancer cell lines.

**Table 1:** The IC_50_ of UA on human cancer cell lines (μM, from MTT assay, 48 h treatment)

Compounds	MDA-MB-231	SW480	MG63	PC3	DU145	HCT116	143B	MDA-MB-468
**UA**	2.669	8.058	19.616	6.026	4.283	5.297	6.220	4.054
**ADR**	0.294	5.782	7.061	0.416	0.150	0.702	0.435	2.323
**5-Fu**	> 500	> 500	> 500	> 500	163.686	> 500	> 500	> 500
**Carb**	> 100	> 100	> 100	> 100	> 100	> 100	37.629	> 100
**CPT**	1.226	5.569	0.265	0.025	0.03	0.147	0.022	0.027
**Taxol**	12.809	38.682	41.107	> 100	< 1E−5	4E−4	4E−4	2.381
**VCR**	8.718	> 100	29.669	> 100	6.27 E−5	1.34 E−4	1.30 E−5	1E−4

### UA exhibits an antiproliferative activity in breast cancer cell lines

As mentioned in previous researches [22–24], UA could inhibit the breast cancer both *in vitro* and *in vivo*. Our result also indicated that UA was shown better inhibition effect on breast cancer cell proliferation. 13 different breast cancer cells were treated with UA or ADR at a range of doses (5 to 60 μM for UA and 0.2 to 10 μM for ADR, Table 2). The cell survival was measured by both MTT and crystal violet assay. MDA-MB-231 cell was found to be the most sensitive cell and SK-BR3 cell was the most resistant to cell death induced by the UA (Table 2).

**Table 2:** Antiproliferation effects of UA on human breast cancer cell lines (IC_50_ (μM) from MTT assay, 48 h treatment)

	UA (μM)	ADR(μM)		UA (μM)	ADR (μM)
**MDA-MB-231**	2.669	0.294	**MCF7**	26.835	7.5
**MDA-MB-361**	7.347	2.074	**67NR**	33.095	6.815
**MDA-MB-435**	20.548	6.934	**4T07**	31.173	3.848
**MDA-MB-468**	4.054	2.323	**4T1**	43.525	7.595
**HS568T**	21.522	4.067	**SK-BR3**	> 60	19.312
**HCC38**	27.657	1.04E-5	**UACC3199**	6.107	3.46
**HCC1937**	17.088	6.734			

UA, at a concentration lower than 50 μM, induced death of the most breast cancer cells that we used here over 48 h of incubation but SK-BR3 cells (Table 2, Supplementary Figure 1A). The data showed that only 2–5 fold higher concentrations of UA were required to induce death in same cells than ADR. These results suggested that UA is a potential agent for the therapy of breast cancer.

### UA induces cancer cell apoptosis and cell accumulation in S phase

As inducing cell apoptosis is a critical mechanism for many anticancer agents, we detected whether UA could induce apoptosis in breast cancer cells. Annex V-EGFP/PI apoptosis assay kit were used to distinguish UA-induced apoptosis from necrosis in 4T1 and MDA-MB-231 cancer cells. Under the staining conditions, we found that UA induced more early apoptotic MDA-MB-231 cells (presented in Q2, 9.29% to 50.37%) and more necrosis in 4T1 cells (presented in Q4) after treated for 24 h (Figure 1B). It indicated that the translocation of phosphatidylserines from the inner surface of cell membrane to the outer surface could effectively increased by UA during the early stages of apoptosis (cells in Q2 quadrant), and ultimately promote the death of tumor cells.

Cell cycle analysis revealed that UA (10 μM) caused a dose-dependent accumulation in S phase in breast cancer cells. The cells in S phase increased from 27.53 ± 5.22% to 52.60 ± 7.79% in MDA-MB-231 cells (24 h), consequently, cells progressed to G_2_ phase were reduced, from 12.99 ± 5.67 in the untreated group to 1.26 ± 1.78 in the treated group (Figure 1C). These findings indicated that UA dramatically blocked cell cycle progression in S phase in 4T1 and MDA-MB-231 cells.

### UA inhibits tumor proliferation of 4T1 tumor bearing mice

4T1 cells labeled with luciferase were implanted to mice to build a stage IV mouse breast cancer model. The bioluminescence imaging model was used for determine the effect of UA on tumor growth. As seen in Figure 1D, animals in control group have higher signal compared to the UA group, the signal of tumors in control group increased from 7.12E + 06 to 1.89E + 08, whereas tumors in UA treated group increased only from 6.84E + 06 to 7.38E + 07 (Figure 1D), which suggested that UA strongly inhibited 4T1 breast tumor growth in mouse model.

**Figure 2:**
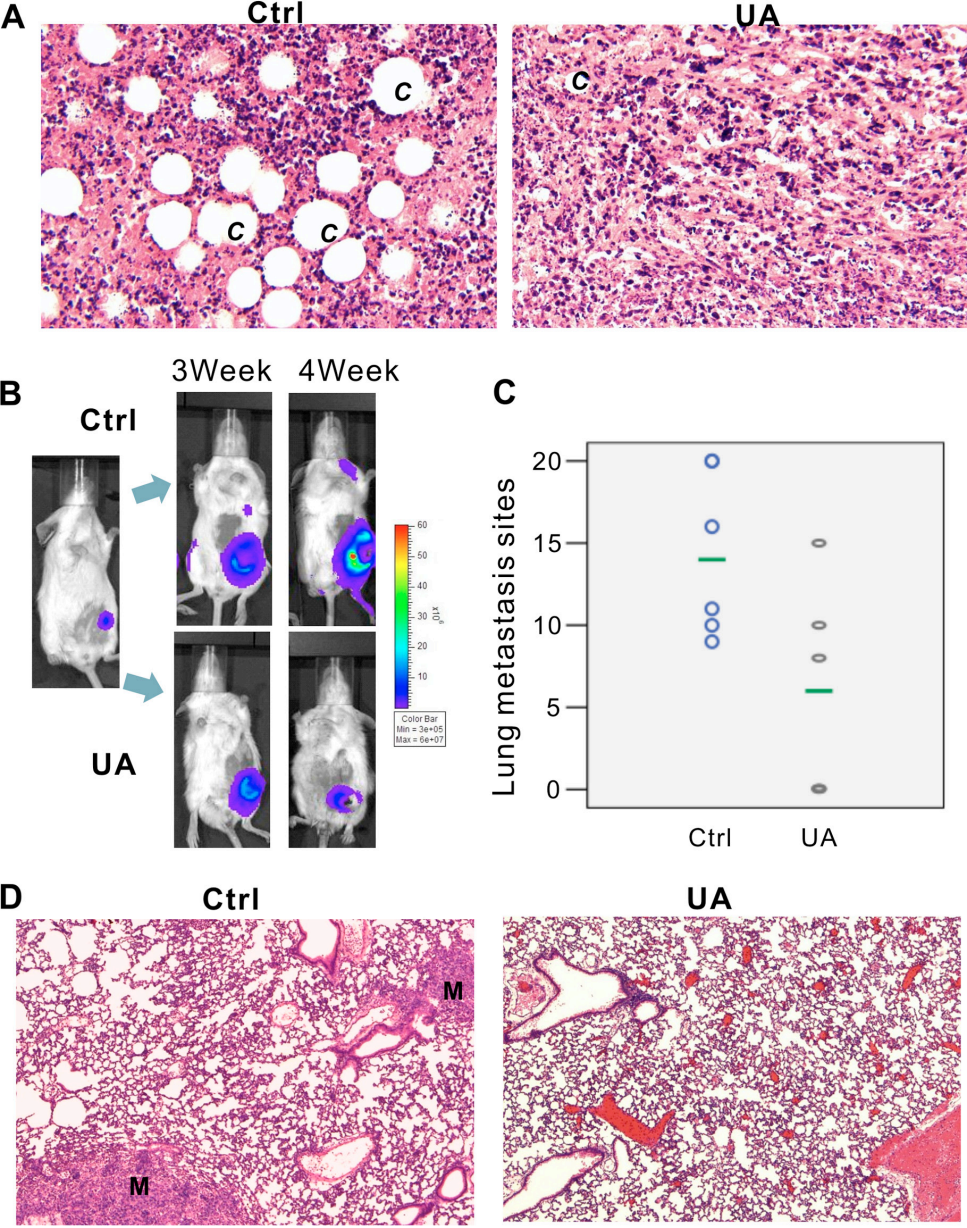
UA inhibits tumor growth and metastasis *in vivo* (**A**) Hemotoxylin & Eosin staining of 4T1 primary tumors (×200 magnification). 4T1-Luc cells were collected and injected into the mammary fat pad of BALB/c mice (5 × 10^5^ cells/injection; six animals per group). Animals were *i.p.* injected with 20 mg/kg UA for 4 weeks and sacrificed. Tumor samples were retrieved for H&E staining. *C* = new capillaries blood vessel in tumors. (**B**) Xenogen images of representative mouse in control group and UA group. Representative Xenogen images of the mice in 3 weeks and 4 weeks are shown. (**C**) UA reduced the number of lung metastasis in 4T1 tumor-bearing mice. Picture shown the number of lung metastasis sites on the lung surface for each mouse. The green line shows the average metastasis for each group. (**D**) Hemotoxylin & Eosin staining of lung tissues for 4T1 tumor-bearing mice and UA treated tumor-bearing mice (×200 magnification). M = metastasis site.

Meanwhile, the average size and the weight of tumors was decreased by UA treatment (Figure 1D and Supplementary Figure 1B), the tumor weight is 1.01 ± 0.14 g in model group and 0.70 ± 0.11 g in UA group (*P* < 0.01). We subsequently conducted H&E staining of the 4T1 tumor tissue samples. The results indicated that tumor cells were slightly less proliferative in UA group but highly proliferative in control group (Figure 2A).

Otherwise, UA at the doses used, was not toxic to the animals as we observed no differences in animal body weights (Supplementary Figure 1C) and behaviors. Histologic examination of tissue sections (liver, kidneys, lung, spleen, brain and heart) from control and UA-treated animals also showed no detectable differences (data not shown).

### UA inhibits lung metastasis of 4T1 tumor bearing mice

In addition to the antitumor growth effect, UA still exhibits the *in vivo* anti-metastasis effectiveness on 4T1 tumor bearing mice (Figure 2B–2D). 4T1 tumor bearing mice produced node metastases (Figure 2B, two -three weeks after MFP injection) and lung metastases (Figure 2B, three-four weeks after MFP injection) in five control mice whereas only two animals in UA group (20 mg/kg/2 days by *i.p.* injection) showed signal in the lung site (Supplementary Figure 2A).

**Figure 3:**
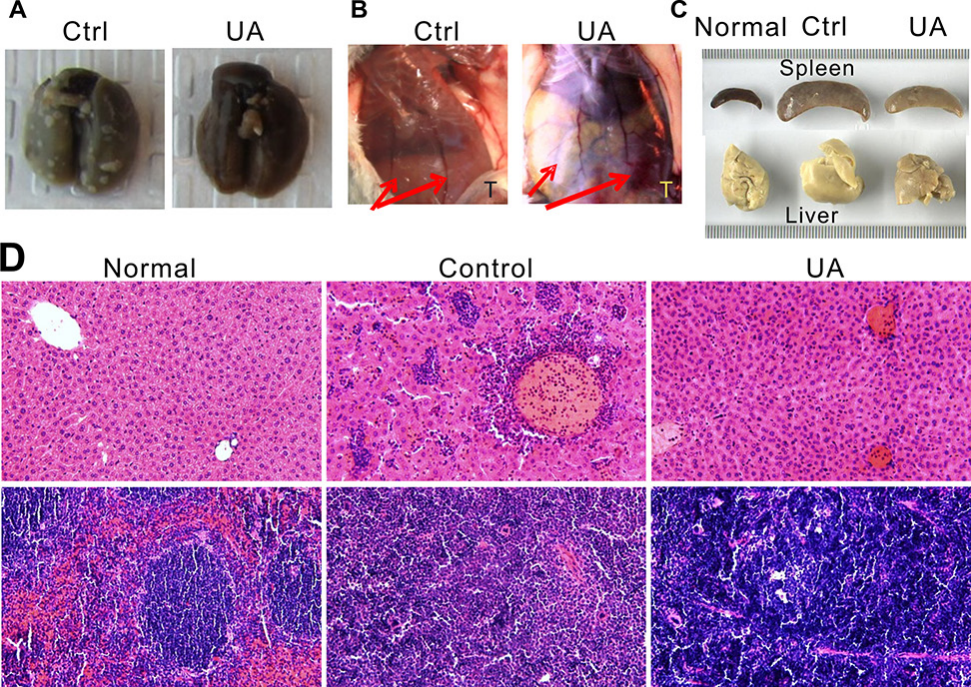
UA suppressed tumor lung metastasis, angiogenesis, leukemoid reaction and extensive extramedullary hematopoiesis of the 4T1 tumor-bearing mice (**A**) UA suppressed lung metastasis. (**B**) The effect of UA on blood vessel diameter. Red arrow indicated the blood vessel of mice; T = tumor site. One representative animal from each group are shown. (**C**) UA relieves liver and spleen enlargement induced by extensive extramedullary hematopoiesis. One representative animal of normal BALB/c mice, 4T1 tumor-bearing BALB/c mice and UA treated tumor-bearing mice are shown. (**D**) Hemotoxylin & Eosin staining of liver (top panel) and spleen (bottom panel) for normal BALB/c mice, 4T1 tumor-bearing mice and UA treated tumor-bearing mice (×200 magnification).

*Ex vivo* image of lung confirmed our bioluminescence results (Figure 3A, one representative animal from each group were shown). After fixation in 10% formalin solution, metastases appear as white nodules on the lung surfaces, so the number and size of visible metastatic lesions on lung surface is easy to identify. The metastasis number on the lung of the controls (6/6 mice have lung metastasis, and the average lung metastasis is 14) and the treatment groups (3/6 mice have lung metastasis, and the average lung metastasis number is 6) are shown in Figure 2C and Supplementary Figure 2B. Presence of metastases in lung were confirmed by H&E, as a result, in most animals of the control group, multiple metastases (M) can be found in lungs after 4 weeks fat pad injection of 4T1 cells, rather than in UA treated group (Figure 2D, one representative animals from each group were shown).

We sought to examine the possible role of UA's anticancer metastatic activity. Tumor cell invasion is the first step for tumor metastasis, so we tested the anti-invasion effects of UA and CAY10585 (HIF-1α inhibitor) on a highly invasive breast cancer cells MDA-MB-231. CAY10585 induced a significant inhibition of MDA-MB-231 cell invasion (61.5% of the control group) through Matrigel coated membrane rather than UA (no significant decrease, Supplementary Figure 2C). The finding indicated that the anti-metastasis effect of UA wasn't related with the MMPs secretion and cell invasion ability.

### UA reduces tumor angiogenesis of the tumor bearing mice

The *in vivo* inhibition effect of UA on angiogenesis was examined in mice model. As shown in Figure 3B, four weeks after 4T1 injection, the diameter of the blood vessels is increased in the tumor implanted side, rather than another side, but after UA treatment, the increase of the blood vessel diameter was significantly inhibited.

The H&E stained breast tumor sections further indicated that inhibition effect of UA on angiogenesis *in vivo*, the new capillaries blood vessel (C) in control group was more than in UA-treated group (Figure 2A). These results demonstrated that UA is a potent inhibitor of vascularization and angiogenesis.

### UA relieves the leukemoid reaction and extensive extramedullary hematopoiesis of the tumor bearing mice

The 4T1 murine mammary carcinoma produces many tumor-derived growth factors, leads to subsequent leukemoid reaction with profound granulocytosis [25]. We found that UA treated mice have less leukemoid reaction than control group (Figure 3D), the liver (Figure 3D, top panel) from tumor-bearing mice in control group revealed numbers of granulocytes in the liver blood vessels and sinusoids but less in the livers from UA treated mice. Otherwise, the WBC and PLT, but not RBC, in mouse peripheral blood also decreased (WBC: 363.49 ± 99.58 10^−9^/L in control *v.s.* 225.88 ± 67.89 10^−9^/L in UA group, *P* < 0.05; PLT:1274.20 ± 58.29 10^−9^/L in control *v.s.* 1054.09 ± 179.89 10^−9^/L in UA group, *P* < 0.05).

Furthermore, this model also displays extensive EMH in mice which also correlated with the expression of tumor-derived chemokines (Figure 3C). In EMH body, hematopoietic elements present in locations other than the bone marrow medullary space. EMH phenomenon may occur in many conditions, such as chronic anemias and blood dyscrasias (leukemia). Spleen and liver are the common sites of EMH. The H&E stained liver (Figure 3C, top panel) and spleen tissues (Figure 3C, bottom panel) indicated that UA could protect the liver and the spleen from the tumor-induced EMH (Figure 3C and 3D).

### Ursolic acid inhibits multiple cell signaling pathways in reporter assay

**Figure 4:**
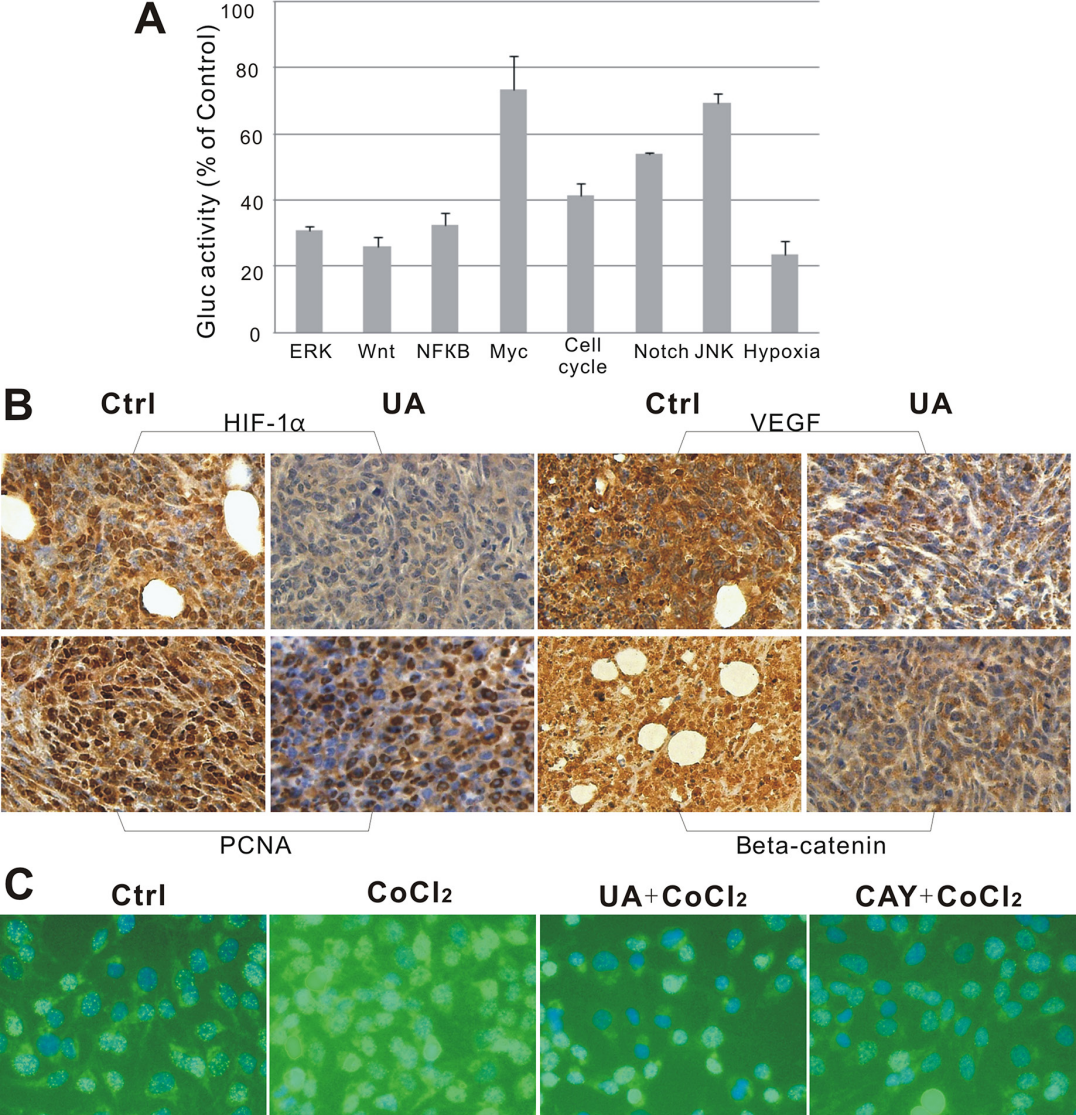
(**A**) Suppressive effect of UA in 8 different signaling pathways. (**B**) Immunohistochemical staining of mice tumor tissues. UA reduced expression of HIF-1α, VEGF, PCNA and β-catenin in tumors. (×400 magnification). (**C**) Immunofluorescence staining of MDA-MB-231 cells. HIF-1α distribution in MDA-MB-231 cells after 24 h treatment with UA (×200 magnification). DAPI (blue) and anti-mouse FITC to detect HIF-1α (green).

We examined the effect of UA on 8 different signaling pathways related with cancer development, including ERK, Notch, Wnt, JNK, NFκB, Myc/Max, pRb-E2F and Hypoxia by reporter assay. When cells were treated with 10 μM UA for 2 h, we found that the luciferase signals of Wnt, Cell cycle, NFκB, Hypoxia and MAPK/ERK was significantly suppressed to less than 50% of the control (Figure 4A), and UA shown the strongest inhibition effects to hypoxia pathway.

### Ursolic acid inhibits HIF-1α, VEGF, PCNA, and β-catenin expression in mouse 4T1 cancer tissue

HIF-1α expression was confirmed in tumor tissues by immunohistochemistry of paraffin embedded tumors slides. We found out that the signal intensity of HIF-1α and its downstream factor-VEGF was significantly decreased by UA after 4 weeks compared with the levels in controls (Figure 4B), which responds to the down-regulation of the hypoxia pathway reporter. Consistent with the IF results seen in Figure 4C, the reduction of HIF-1α in MDA-MB-231 cells by UA was seemingly caused by decreasing the nuclear staining intensity of positively stained cells compared to that of the CoCl_2_ (a chemical inducer of HIF1) group.

For the PCNA expression in the cell nuclei during the DNA synthesis phase of the cell cycle is under the control of pRb-E2F transcription factor-containing complexes, we further analyzed the expression of PCNA in the nuclear. The result indicated that cells with high PCNA expression were significantly decreased in UA group (Figure 4B).

From our reporter assay results, the Wnt pathway was down-regulated and the activity of TCF/LEF was reduced by UA treatment. As we know, β-catenin is involved in the transduction of Wnt pathway and the nuclear level of β-catenin is associated with the transcriptional regulation of the transcription factors in TCF/LEF family. So the endogenous level of total β-catenin protein in the tumor tissue was detected, and the staining intensities of β-catenin in whole-cell and nuclear were markedly reduced by UA compared with that of the tumors from the control group (Figure 4B).

### UA upregulated VHL gene expression in MDA-MB-231 cells

**Figure 5:**
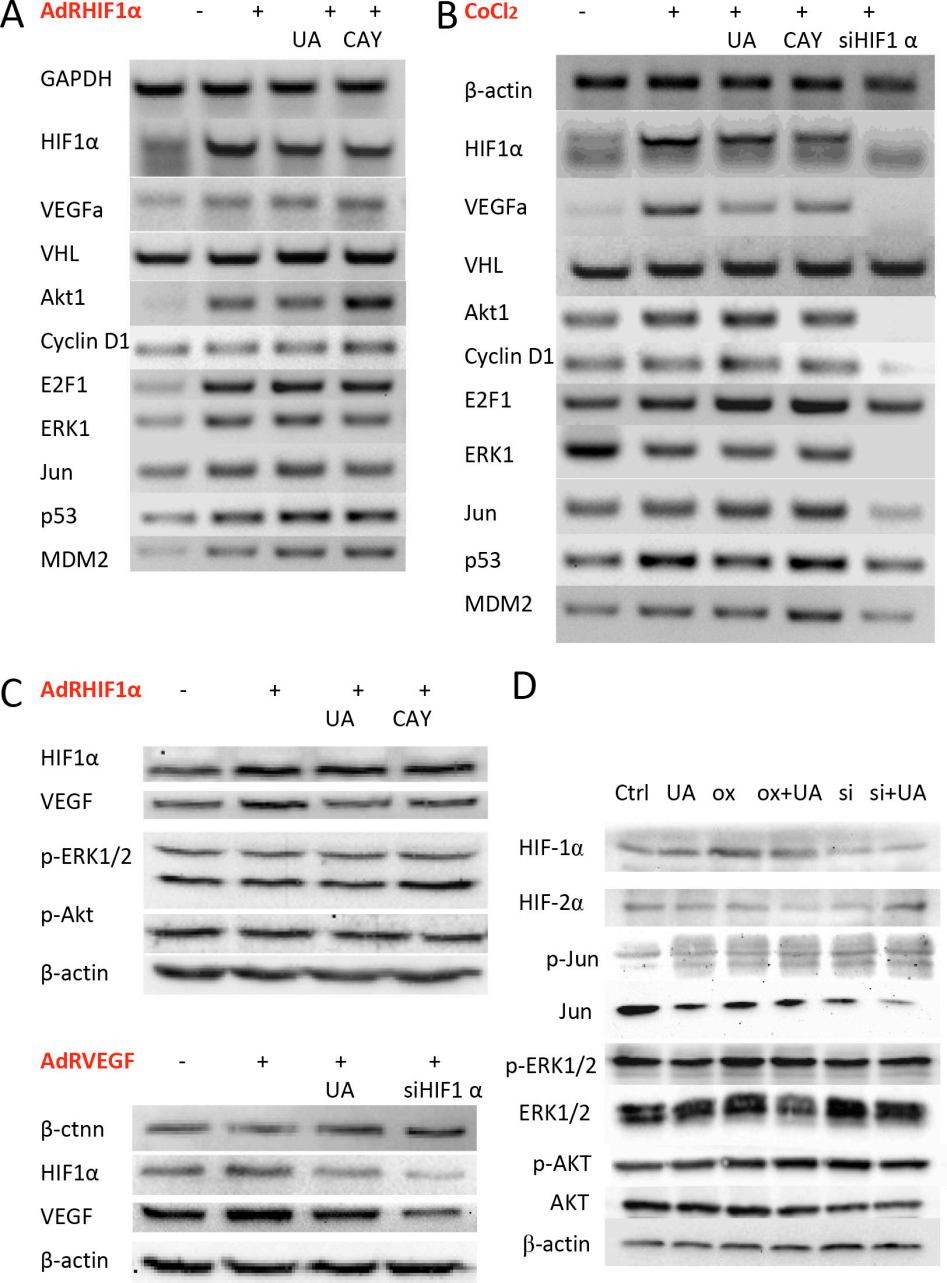
Effect of UA on HIF-1α and VEGF expression in breast cancer cells (**A**) The mRNA expression levels of HIF-1α, VEGF and related factors in MDA-MB-231 cells after HIF-1α over expressed. (**B**) MDA-MB-231 cells were treated with CoCl_2_ (a chemical inducer of HIF1). (**C**) The protein expression levels of HIF-1α and VEGF on HIF-1α or VEGF over expressed MDA-MB-231 cells were examined by western blotting. (**D**) The related protein levels in HIF-1α over expressed 4T1 cells.

To further determine if UA shows specificity inhibition effects to hypoxia pathway, the HIF-1α, VHL, GAPDH, β-actin and VEGFα mRNA level was measured by RT-PCR. OxHIF-1α or RFP for 48 h and then treated with or without UA and CAY10585 for 24 h, the HIF-1α level of UA or CAY10585 group was decreased significantly when compared with that of AdRHIF-1α group (Figure 5A). In contrast, the UA or CAY10585–treated cells markedly enhanced in VHL (tumor suppressor) levels [26] compared with the AdRHIF-1α group (Figure 5A). We also found the HIF-1α and VEGF levels of UA or CAY10585 treated and AdRsiHIF-1α infected cells was decreased significantly when compared with the levels in CoCl_2_ group. Thus, UA could inhibit HIF-1α-VEGF pathway by inducing VHL expression and improve the HIF-1α degradation.

### UA changed E2F1, p53 and MDM2 expression in breast cancer cells with HIF-1α overexpression

Next, cell proliferation related factors were also detected by RT-PCR. Previous research indicated that mice deficient in E2F1 has more angiogenesis than normal mice [27] and overexpression of E2F1 inhibited the activity of VEGF promoter which induced by hypoxia in cancer cells. E2F1 also stabilized p53 protein and then suppressed VEGF expression and neovascularization [28]. Our results (Figure 5B) shown UA treated HIF-1α overproduction cells have high E2F1 transcription level, indirectly affected the levels and activity of p53 [29]. These results indicated that E2F1, p53 and MDM2 were all involved in the antitumor angiogenesis and metastasis effects of UA.

### UA decreased HIF-1α and VEGF protein level in human breast cancer cell MDA-MB-231

The western blotting result exhibits the similar results in HIF-1α and VEGF's protein levels. Adenoviruses expression of HIF-1α (AdRHIF-1α) or VEGF (AdRVEGF) for 48 h resulted in the overexpression of HIF-1α and VEGF proteins in MDA-MB-231 cells, and treated with UA or CAY10585 for 24 h could reduce the HIF-1α and VEGF expression in the AdRHIF-1α or AdRVEGF infected cells (Figure 5C). In 4T1 cells with HIF-1α overexpression, UA slightly decreased the expression of HIF-1α, HIF-2α and ERK, the expression of Jun, Akt and p-Akt did not change significantly (Figure 5D).

### Breast cancer cells with HIF-1α over expressed are more resistant to UA-induced antiproliferative activity

**Figure 6:**
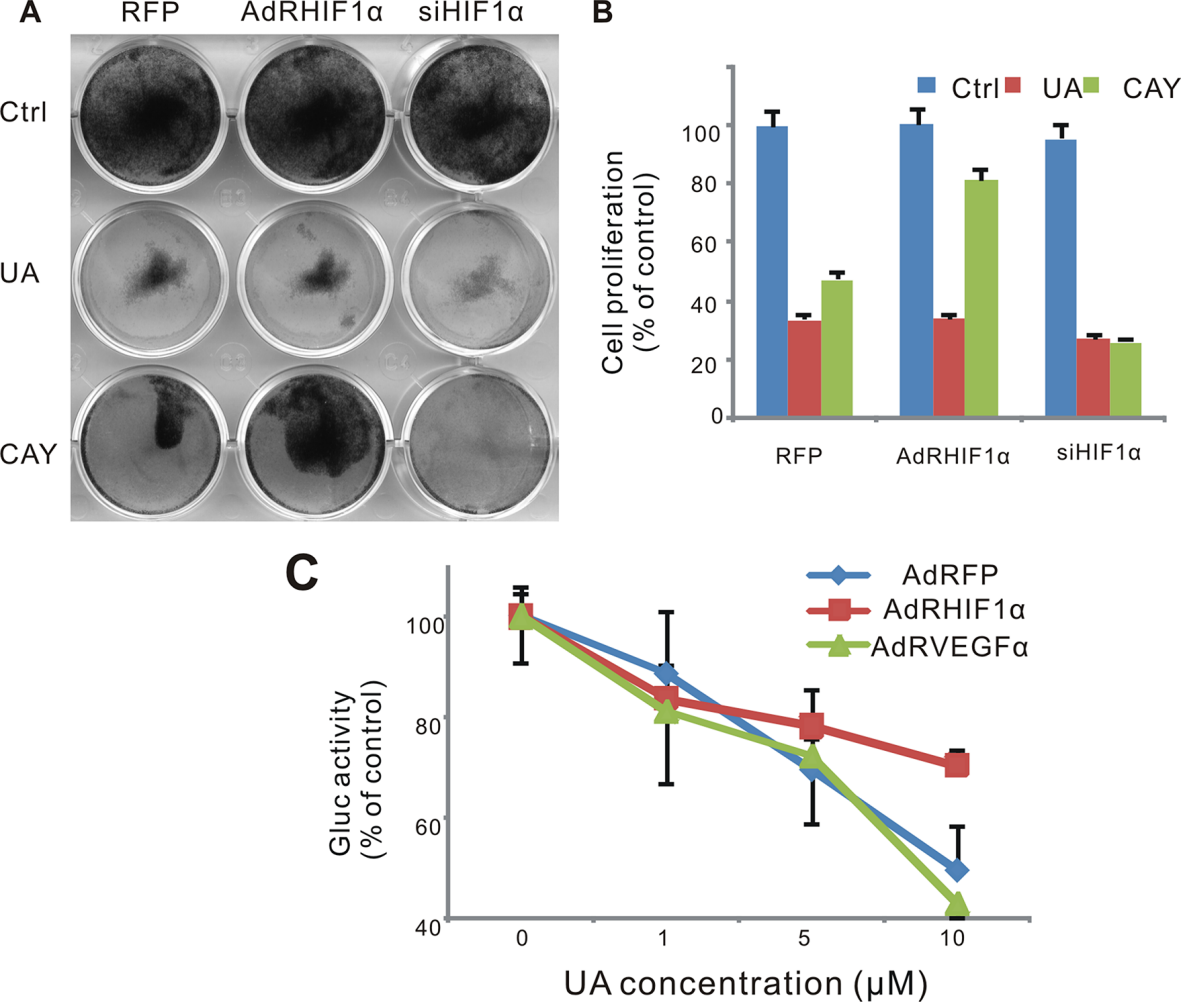
Effect of UA in hypoxia pathway in MDA-MB-231 cells (**A**) Cell was infected with AdRFP, AdRHIF-1α or AdRsiHIF-1α virus for 16 h, followed by UA treatment in 1% FBS DMEM medium for 48 h, cell survival was tested by crystal violet assay. (**B**) Cell stained with crystal violet (show in Figure 6A) were dissolved with 20% acetic acid and measured at 570 nm. (**C**) MDA-MB-231 -HIF-1α-GLuc cells were infected with AdRFP, AdRHIF-1α or AdRVEGF and treated with UA in serum free DMEM medium for 2 hour. The Gaussia luciferase (GLuc) activity of the cell medium was measured by the Gaussia Luciferase Assay kit. The inhibition was presented as the percentage of the control group.

To validate the role of HIF-1α in the antitumor effect of UA, we tested the cell survival after UA treatment when HIF-1α was over expressed or silenced. As a result, MDA-MB-231 cells were sensitive to UA treating after the silencing of HIF-1α (Figure 6A and 6B). The response of HIF-1α pathway reporter to UA was suppressed when HIF-1α was over expressed but increased when VEGF was over expressed, which suggested that VEGF induced HIF-1α activity could be blocked by UA and VEGF also play an important role in the antitumor effect of UA (Figure 6C).

## DISCUSSION

Some previous studies have shown that UA can inhibit several cancer cells through multiple pathways[30, 31], namely NF-κB [32], ASK1-JNK [33], STAT3 [34], JNK [35] and TGF-β1/miR-21/PDCD4 Pathways [36], and so on. Our reporter assay also exposed that several pathways, including HIF-1α, ERK, TCF, NF-κB, and Elk, were changed by UA treatment, among which HIF-1α was the most significant reduced (up to about 76% suppression).

Hypoxic condition in tumor mass is thought to be one trigger for cancer metastasis, it could activate multiple signaling pathways that stimulate imbalance hematopoiesis and cancer cells invasiveness [30]. HIF-1α binds to HREs in genes such as VEGF and erythropoietin (EPO) leading to imbalance hematopoiesis and angiogenesis [37]. As we known, HIF-1α and VEGF/Flt1 autocrine loop also plays an important role in the hypoxia-mediated drug resistance [38]. In addition, aberrant VEGF and EPO expression closely associated with tumor-induced EMH [11, 39]. Here, the protein and mRNA levels of VEGF were tested to confirm the inhibition effect of UA on cancer angiogenesis and EMH. We found UA blocked the hypoxia-driven VEGF/Flt1 autocrine survival pathway by directly depressed the mRNA level of HIF-1α and VEGF gene, and then decreased protein expression of HIF-1α and VEGF. Earlier studies focused on the relationship between UA and VEGF also demonstrated the VEGF expression and activity were reduced by UA treatment, which could attenuate angiogenic and metastatic actions of cancer cells [21, 30].

On the other hand, recent investigations suggest that, the tumor suppressor VHL mutation in mice enhanced expression of key HIF-2α genes, then promoted splenic erythropoiesis [9]. In normoxic conditions, VHL activates an ubiquitin-dependent proteasome system through specifically interacts with hydroxylated HIF [26, 37]. Therefore, HIF-1α is ubiquitinated and subsequently degradated when the VHL is high expressed in cells. We notified that UA increases VHL mRNA expression in breast cancer cells, which leads a low level of HIF-1α and HIF-2α.

HIF-1 also plays a critical role in p53 accumulation [28, 29]. Several reports support the hypothesis that HIF-1α induced the cell death in hypoxic and/or glucose deprivation conditions, possibly by associating with prevention the degradation of p53 [37]. Thus, the p53 and MDM2 expression also serves the tumor cells to survive hypoxia, and recruits endothelial cells at the early stage of angiogenesis. We have shown that UA can inhibit the p53 and MDM2 gene expression in HIF-1α overproduction MDA-MB-231 cells.

Further understanding of the E2F1 gene expression indicated that the E2F1 also play a critical role between VEGF-HIF-1α loop and the p53-MDM2 activity. Previous study shows that E2F1 could stabilize p53 protein to suppress VEGF expression. E2F1-deficient mice show a dysregulation of VEGF and then might increase vascular supply of tumors. [27, 28]. Our RT-PCR results presented a high level of E2F1 mRNA level in hypoxia MDA-MB-231 cells. UA induced both E2F1 and p53 expression. That could lead to more effective treatment strategies for tumor proliferation and angiogenesis.

**Figure 7:**
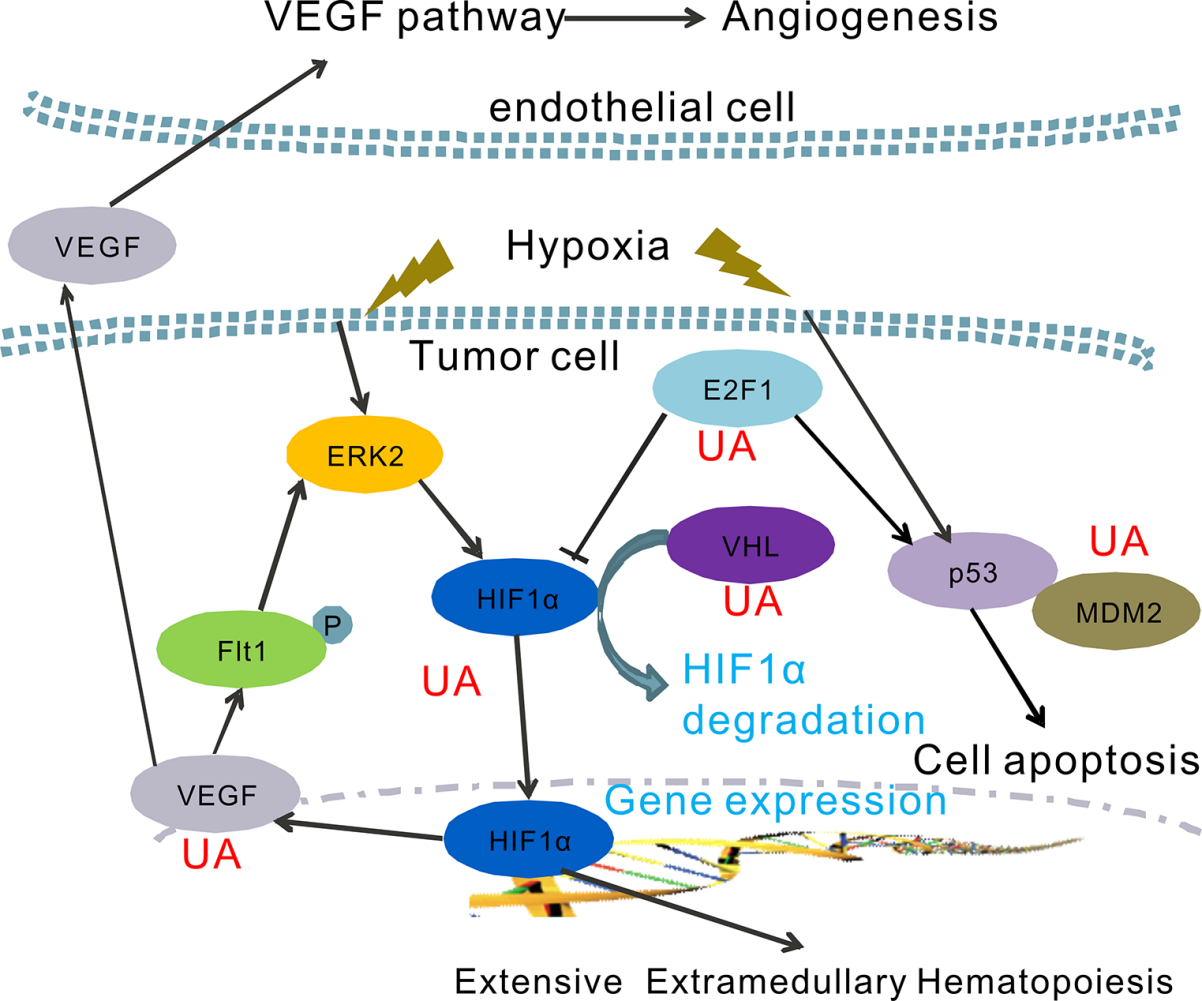
A hypothetical model of the functions of UA on the regulation of hypoxia pathway UA inhibits HIF-1α-VEGF pathway by inducing VHL expression and improve the HIF-1α degradation.

In summary, we report a naturally anti-metastatic agent, ursolic acid. The results presented in this paper support that the anticancer activity of UA may be partially caused by blocking the hypoxia signaling pathway in cancer. Together with the data presented here, many factors, namely HIF-1α, HIF2α, VEGF, VHL, E2F1, p53 and MDM2 were involved in the anti-EMH and metastasis effects of UA. The exact mechanism of action of UA on the inhibition of HIF-1α activity and VEGF expression in cancer cells is proposed in Figure 7.

Conclusively, we have demonstrated in this study that UA effectively inhibits tumor growth and metastasis of 4T1 breast tumor bearing BALB/c mice. Our findings indicate that UA has a promising anticancer effect, and its anticancer activity may be at least in part related with the blocking of cancer hypoxia and hypoxia-driven EMH and metastasis.

## MATERIALS AND METHODS

### Cell culture

Human cancer lines SW480 and HCT116 (colorectal cancer), MDA-MB-231 and MDA-MB-468 (breast cancer), PC3 and DU145 (prostate cancer), MG63 and 143B (osteosarcoma), human embryonic kidney-293 (HEK293) cells, as well as mouse breast cancer 4T1 and 4T07 cell, were purchased from the American Type Culture Collection (Manassas, VA), other human breast cancer cell lines 67NR, MCF7, UACC3199, HCC38, HCC1937, MDA-MB-361, MDA-MB-435, SK-BR-3 and HS578T were provided by Dr. Kathy Goss. All of the cells were grown in RPMI-1640 or Dulbecco's modified Eagle's medium (Invitrogen, Carlsbad, CA) supplemented with 10% fetal bovine serum (HyClone Laboratories, Logan, UT) and 50 U penicillin/streptomycin in 5% CO_2_ at 37°C.

### Chemicals and drug preparations

Ursolic acid, ADR, and vincristine were purchased from Sigma-Aldrich (St. Louis, MO). Camptothecin, carboplatin, CAY10585 (HIF-1α inhibitor) and 5-fluorouracil were obtained from ENZO Life Sciences (Plymouth Meeting, PA); and taxol was purchased from Santa Cruz Biotechnology (Santa Cruz, CA). These compounds were dissolved in DMSO to make stock solutions and were kept at −80°C as aliquots. Unless otherwise indicated, other chemicals were from Fisher Scientific (Waltham, MA) or Sigma-Aldrich (St Louis, MO, USA).

### Recombinant adenoviruses expressing RFP, HIF-1α, SiHIF-1α and VEGF

Recombinant adenoviruses were generated using AdEasy technology as described [40]. The coding regions of human HIF-1α and human VEGF were PCR amplified and cloned into an adenoviral shuttle vector and subsequently used to generate recombinant adenoviruses in HEK293 cells. Similarly, short interfering RNA (siRNA)-coding oligonucleotides against human HIF-1α were designed by using Dharmacon's siDESIGN program. The oligonucleotide cassettes were cloned into pSES shuttle vector as previously described [41, 42]. All constructs were verified by DNA sequencing. Recombinant adenoviruses, designated as AdRHIF-1α, AdRVEGF and AdR-siHIF-1α were generated as previously described [40, 43]. AdRHIF-1α, AdR-siHIF-1α, and AdRVEGF express RFP as a marker for monitoring infection efficiency. Analogous adenovirus expressing only monomeric RFP (AdRFP) was used as control.

### Pathway pBGLuc reporter constructions and stable cell line establishment

All the reported constructions of the transcription factors for signaling pathways MAPK/ERK, MAPK/JNK, Wnt, Notch, Cell Cycle/pRb-E2F, nuclear factor κB (NFκB), Myc/Max and Hypoxia, namely eag-like K^+^ channel /serum response factor (Elk-1/SRF), activator protein 1 (AP-1), TCF/LEF, RBP-Jκ, E2F/DP1, NFκB, Myc/Max and HIF-1, were cloned into our pBGLuc vector, respectively. All construes were sequenced to confirm mutation. Human cancer line HCT116 was stably transduced with these 8 reporters as described previously [44]. Additionally, the murine breast cancer 4T1 was stably transduced with firefly luciferase by using a retroviral vector expressing firefly luciferase as described previously [45]. In brief, recombinant retrovirus was packaged in HEK293 cells by co-transfecting cells with pSEB-Luc and pAmpho packaging plasmid using LipofectAMINE (Invitrogen). Pooled stable cells were selected with blasticidin S (6–8 μg/ml) for 4 days. Firefly luciferase and Gaussia luciferase activity was confirmed by using the Luciferase Assay Kit (Promega, Madison, WI) or Gaussia Luciferase Assay Kit (New England Biolabs).

### MTT proliferation assay

A modified MTT assay was used to examine the cell proliferation as described previously [46]. In brief, cells were seeded in 96-well plates (10^4^ cells/well, 50–70% density). Drugs were added to the cells at variable concentrations or solvent control. After 48 h treatment, 15 μl of MTT dye solution was added to each well and incubated for an additional 4 h. Thereafter, cell culture medium was removed and 100 μl/well DMSO was added to dissolve formazan crystals at room temperature for 10–15 min. Absorbance at 570 nm was measured using a 96-well microplate reader.

### Crystal violet viability assay

Crystal violet assay was conducted as described previously [45]. Cells were treated with drugs. After 48 h treatment, cells were carefully washed with PBS and stained with 0.5% crystal violet formalin solution at room temperature for 30 min. The stained cells were washed with tap water and air dried for taking images. For quantitative measurement, the stained cells were dissolved in 20% acetic acid (1 ml per well for 12-well plate) at room temperature for 20 min with shaking. A 250 μl solution was taken and added to 1 ml of H_2_O. Absorbance was measured at 570 nm.

### Boyden chamber trans-well cell invasion assay

The experiments were carried out as described previously [47, 48]. Subconfluent MDA-MB-231 cells were trypsinized and washed in Dulbecco's modified Eagle's medium twice. Pre-equilibrated media containing 1% fetal bovine serum as a chemoattractant was placed into the bottom chamber of 24-well transwell unit (BD Biosciences, San Jose, CA). Cells (5 × 10^4^) were placed onto each upper chamber of the transwell unit, in which the polycarbonate 8 μm pore membrane was precoated with 10 μg matrigel mix (BD Biosciences, San Jose, CA) for 4 h and washed in PBS. Cells were allowed to migrate at 37°C and 5% CO_2_ for 24 h. Cells on the upper side were gently wiped off with a wet cotton-tip applicator, and the membrane containing attached cells was fixed in 10% formalin and stained with DAPI. The membranes were mounted onto slides with Permount (Thermo Fisher Scientific, Waltham, MA). The number of migrate cells per high-power fields was determined by Image-Pro Plus 5.1 software. The assays were performed in duplicate and were reproducible in at least two batches of independent experiments.

### Annexin V-EGFP/PI apoptosis assay

MDA-MB-231 cells were seeded in 6-well plates and treated with UA at various concentrations for 24 h. 1 × 10^5^ Cells were washed with PBS twice and incubated with 200 μl of binding buffer, 2 μl of PI (20 ng/ml) and 2 μl of Annexin V-EGFP fusion protein (GenScript USA Inc., Piscataway, NJ) in each well for 15 min at room temperature, followed by flow cytometry analysis. Each assay condition was performed in triplicate.

### Cell cycle analysis by flow cytometry

Flow cytometry was used to quantitatively detect the cell-cycle distribution [49]. Cells (1 × 10^5^ /well) were plated into 6 well plates 1 day before treatment with UA at various concentrations. After treatment for 24, 48 and 72 h, cells were harvested, washed with PBS, fixed in cold Methanol overnight at 4°C for at least 2 h, and stained with 50 ng/ml propidium iodide (PI) by incubation at 4°C for at least 15 min. The stained cells were analyzed by flow cytometry (Becton–Dickinson).

### Immunofluorescence staining

Cultured cells were treated with UA for 24 h, and then fixed with cool methanol and washed with PBS. The fixed cells were permeabilized with 0.5% Triton-100 and blocked with 5% BSA, followed by incubation with an anti-HIF-1α antibody (Santa Cruz, CA, USA) for 4 hour. After washing, cells were incubated with anti-mouse FITC secondary antibody for 30 minutes, followed by incubating cells with DAPI for 10 minutes at room temperature.

### Transfection and reporter assay

Gaussia luciferase reporter assay was carried out as described by protocol. In brief, 2 × 10^4^ /well HCT116 cells (transduced with the 8 pathway reporters) were seeded in 96 well plate and treatment with UA at various concentrations in serum free DMEM medium. After 2 h treatment, cell medium was subjected to luciferase activity assays using BioLux^®^ Gaussia Luciferase Assay Kit (New England Biolabs) [50].

### RNA isolation and semi-quantitative RT-PCR analysis

Total RNA was isolated using TRIZOL Reagents (Invitrogen). Total RNA was used to generate cDNA templates by RT reaction with hexamer and Superscript II RT (Invitrogen). The first strand cDNA products were further diluted 10-fold and used as PCR templates. Semiquantitative RT-PCR was carried out as described previously [41]. PCR primers were designed by using the Primer3 program to amplify the genes of interest (approximately 150–180 bp). Primer sets used for these analyses are listed in Supplementary Table 1. The specificity of PCR products was confirmed by resolving on 1.5% agarose gels. All samples were normalized by the expression level of GAPDH.

### Protein extraction and western blotting analysis

Western blotting was performed as previously described [51, 52]. Briefly, cells were collected and lysed in RAPI buffer. Cleared total cell lysate was denatured by boiling and loaded onto a 10% gradient SDS–PAGE. After electrophoretic separation, proteins were transferred to an Immobilon-P membrane. Membrane was blocked with SuperBlock Blocking Buffer, and probed with the primary antibody, anti-HIF-1α, anti-VEGF, anti-p-Akt, anti-β-actin (Santa Cruz, CA) and anti-p-ERK1/2 (Cell Signaling Technology, Vancouver, Canada), followed by incubation with a secondary antibody conjugated with horseradish peroxidase. The proteins of interest were detected by using SuperSignal West Pico Chemiluminescent Substrate kit.

### 4T1 tumor bearing mice model and xenogen bioluminescence imaging

The use and care of animals was carried out by following the guidelines approved by the Institutional Animal Care and Use Committee. Subconfluent 4T1-Luc cells were harvested and resuspended in PBS to a final density of 1 × 10^7^ cells/ml. Before injection, cells were resuspended in PBS and analyzed by 0.4% trypan blue exclusion assay (viable cells, > 90%). For cancer cell injection, approximately 5 × 10^5^ 4T1-Luc cells in 50 μl of PBS were injected into the mammary fat pad (MFP) of each female BALB/c mouse (Harlan, Indianapolis, IN) (4 weeks old, 18–20 g) using 27-gauge needles (*n* = 6) [25]. At 48 h after tumor cell injection, UA was administered at 20 mg/kg body weight to mice once every 2 days via intraperitoneal (*i.p.*) injection.

Small animal whole-body optical imaging was carried out as described previously [45]. In brief, mice were anesthetized with isoflurane attached to a nose-cone mask equipped with Xenogen IVIS 200 imaging system (Caliper Life Sciences, Hopkinton, MA) and subjected to imaging weekly after MFP injection. For imaging, mice were injected intraperitoneally with D-luciferin sodium salt (Gold Biotechnology, St. Louis, MO) at 100 mg/kg body weight in 0.1 ml of sterile PBS. Acquired images were obtained by superimposing the emitted light over the grayscale photographs of the animal. Quantitative analysis was done with Xenogen's Living Image V2.50.1 software as described previously [53]. Animals were taken *in vivo* images weekly for both untreated and treated groups and sacrificed after 4 weeks. 100 μL of mouse whole blood was collected (EDTA as *in vitro* anticoagulant) before sacrificed. Routine blood test was immediately performed on a Sysmex XT-2000i automated haematology analyser (sysmex, Japan) for the white blood cells (WBC), red blood cells (RBC), and platelets (PLT) et al. Tumor samples were retrieved for histological examination.

### Histological evaluation and immunohistochemical(IHC) staining

Retrieved tumor tissues were fixed in 10% formalin and embedded in paraffin. Serial sections of the embedded specimens were stained with hematoxylin and eosin (H&E). For immunohistochemical staining, slides were deparaffinized and then rehydrated in a graduated fashion. The deparaffinized slides were subjected to antigen retrieval and probed with anti-PCNA, anti-HIF-1α, or anti-VEGF antibody (Santa Cruz Biotechnology) or isotype IgG control, followed by incubation with biotin secondary antibodies and streptavidin-horseradish peroxidase. The presence of the expected protein was visualized by DAB staining and examined under a microscope. Stains without the primary antibody or with control IgG were used as negative controls [54].

### Statistical analysis

All quantitative experiments were performed in triplicate and/or repeated three times. Data were expressed as mean ± S.D. Statistical significances between vehicle groups versus drug-treatment groups were determined by one-way analysis of variance and the Student's *t* test. A value of *p* < 0.05 was considered statistically significant.

## SUPPLEMENTARY MATERIALS FIGURES AND TABLES



## Abbreviations

ADR: adriamycin also known as adriamycin
AP-1: activator protein 1
ASK1: apoptosis signal- regulating kinase1
BLI: bioluminescence imaging
DAPI: 4'6-diamidino-2-phenylindole
DMSO: dimethyl sulphoxide
Elk: eag-like K+ channel
EMH: extramedullary hematopoiesis
ERK: extracellular signal-regulated kinase
Flt1: fms-like tyrosine 1
GAPDH: glyceraldehyde-3-phosphate dehydrogenase
H&E: hematoxylin and eosin
HIF: hypoxia-inducible factor
IHC: immunohistochemical
JNK: c-Jun N-terminal kinase
LEF: lymphoid enhancer binding factor
MDM2: MDM2 proto-oncogene
MFP: mammary fat pad
Myc/Max: v-myc avian myelocytomatosis viral oncogene homolog/MYC associated factor X
NF-κB: nuclear factor-κ-gene binding
p53: protein 53
PCNA: proliferating cell nuclear antigen
PDCD4: programmed cell death 4
PI: propidium iodide
PLT: platelets
RBC: red blood cell
RBP-Jκ: recombination signal binding protein Jκ
SRF: serum response factor
STAT3: signal transducer and activator of 3
TCF: transcription factor
TGF-β1: transforming growth factor-β1
UA: ursolic acid
VEGF: vascular endothelial growth factor
VHL: von hippel-lindau
WBC: white blood cell.
