# Supplementary material for: Hypoxia pathway and hypoxia-mediated extensive extramedullary hematopoiesis are involved in ursolic acid's anti-metastatic effect in 4T1 tumor bearing mice
Source: Oncotarget. 2016 Sep 30;7(44):71802–16. doi: 10.18632/oncotarget.12375 (PMC5342124; doi:10.18632/oncotarget.12375)
Supplement: Supplementary file 1 [file oncotarget-07-71802-s001.pdf]

Hypoxia pathway and hypoxia-mediated extensive extramedullary hematopoiesis are involved in ursolic acid's anti-metastatic effect in 4T1 tumor bearing mice

Supplementary Materials

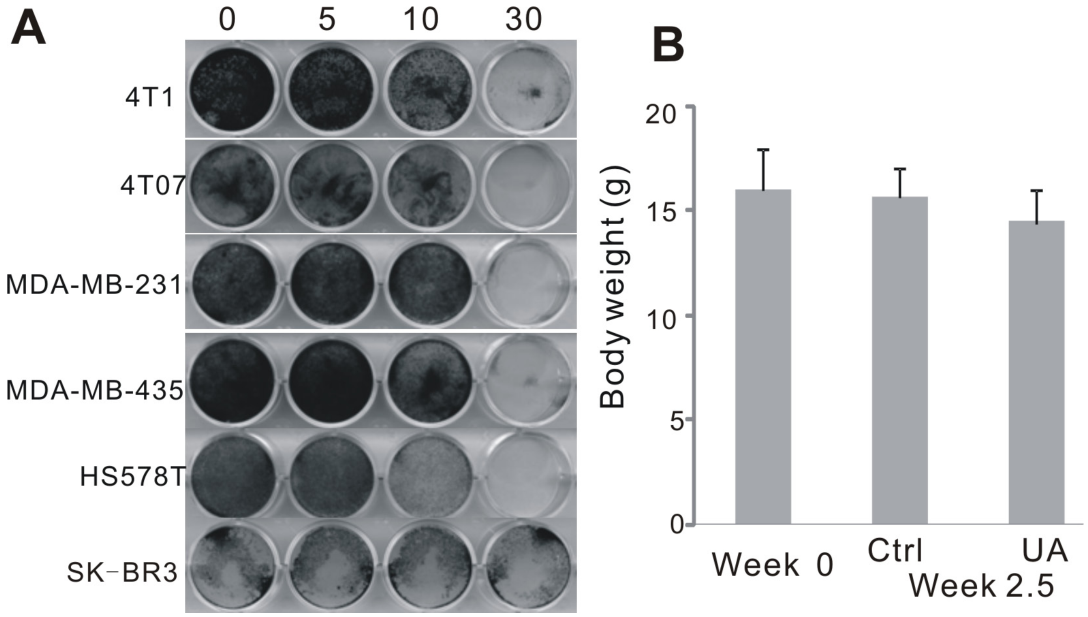

Supplementary Figure S1: (**A**) Effect of UA on the proliferation of breast cancer cells. Different kinds of breast cancer cells were treated with designed concentration for 48 hours and stained with crystal violet. (**B**) The effect of UA on mice body weight after 18 days treatment.

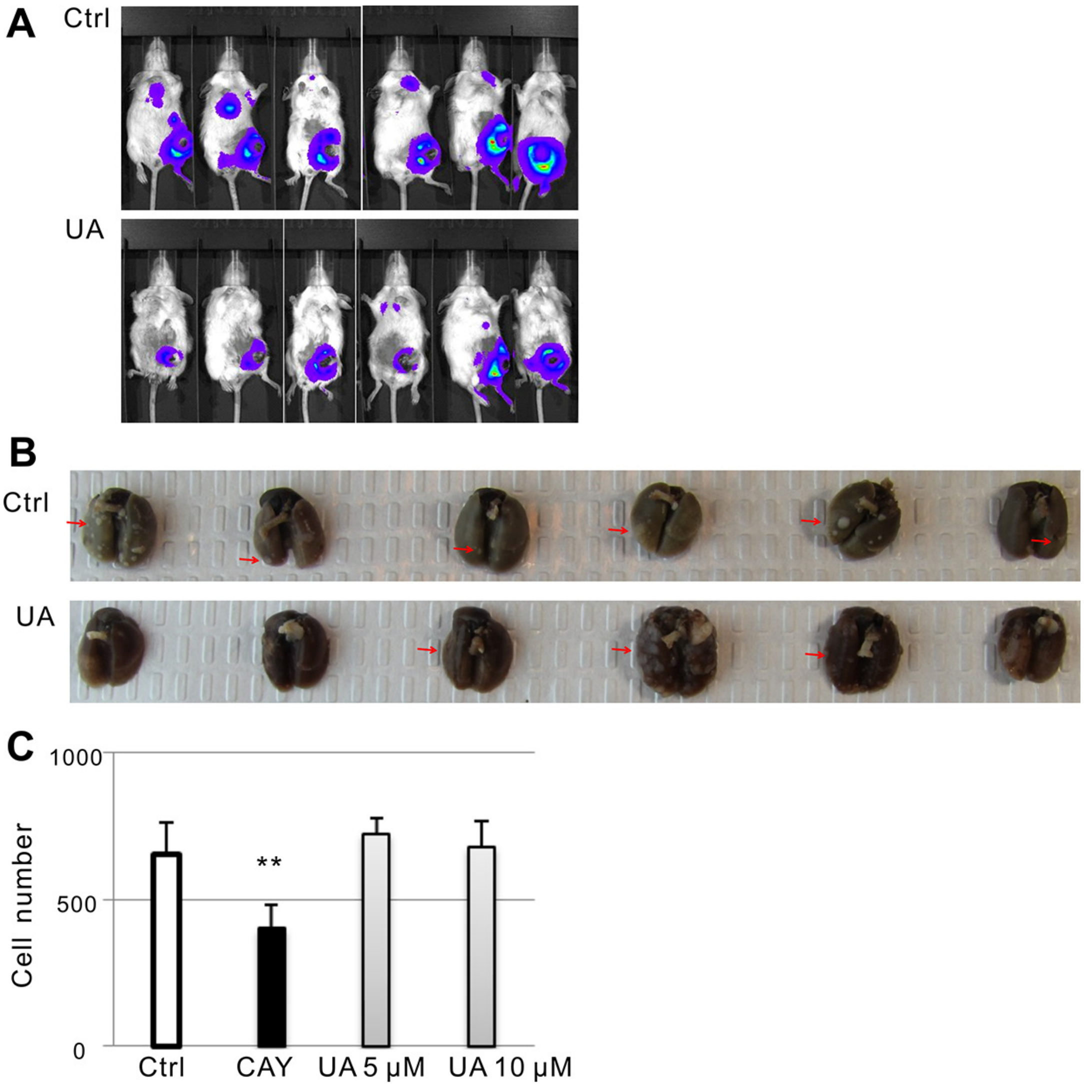

Supplementary Figure S2: Effect of UA on tumor growth and metastasis *in vivo*. (**A**) The Xenogen images of control group and UA treated group mice after 4 weeks UA treatment. UA suppressed the lung metastasis (top panel, control; bottom panel, UA group). Briefly, luciferase-tagged 4T1 cells were collected and injected into the mammary fat pad of BALB/c mice (0.5×10^5 cells/injection; six animals per assay condition). Animals were treated with 20 mg/kg UA and subjected to Xenogen imaging weekly. (**B**) The effect of UA on lung metastasis (control, top panel; UA group, bottom panel). Red arrow indicated the lung with metastasis; one representative animal from each group are shown. (C) UA didn't affect the cell invasion in MDA-MB-231 cells *in vitro*.

Supplymentary Table S1: Primer sequences of target genes and housekeeping gene for RT-PCR

Gene	Forward Primer	Reverse Primer
**human HIF1alpha**	TGCATCTCCATCTCCTACCC	CGTTAGGGCTTCTTGGATGA
**human VEGF a**	CCCACTGAGGAGTCCAACAT	TTTCTTGCGCTTTCGTTTTT
**human VHL**	GGTGGCATTTTTGCTTCCTA	CAGTCTTCCCAAAGCAGGAG
**human p53**	GGCCCACTTCACCGTACTAA	GTGGTTTCAAGGCCAGATGT
**human Cyclin D1**	TGTTTGCAAGCAGGACTTTG	TGGCACCAAAGGATTCCTAA
**human E2F1**	ATGTTTTCCTGTGCCCTGAG	ATCTGTGGTGAGGGATGAGG
**human Jun**	CAGGTGGCACAGCTTAAACA	TTTTTCTCTCCGTCGCAACT
**human MDM2**	CCGAATAAGGTTTGCCTGAA	CAAATTGCAAAAGGCACTGA
**human AKT1**	AGAAGCAGGAGGAGGAGGAG	TCTCCTTCACCAGGATCACC
**human ERK1**	CCAGACCATGATCACACAGG	CTGGAAAGATGGGCCTGTTA
**human GAPDH**	CAGCGACACCCACTCCTC	TGAGGTCCACCACCCTGT

HIF1alpha, hypoxia-inducible factor 1 alpha; VEGF a, vascular endothelial growth factor a; VHL, von Hippel-Lindau; p53, protein 53; MDM2, murine double minute 2; AKT1, thymoma viral proto-oncogene 1; ERK1, extracellular regulated protein kinases 1; GAPDH, glyceraldehyde-3-phosphate dehydrogenase.
